# Resilience Assessment Scale for the Prediction of Suicide Reattempt in Clinical Population

**DOI:** 10.3389/fpsyg.2021.673088

**Published:** 2021-05-13

**Authors:** David Sánchez-Teruel, María Auxiliadora Robles-Bello, José Antonio Muela-Martínez, Ana García-León

**Affiliations:** ^1^Department of Psychology, University of Cordoba, Cordoba, Spain; ^2^Department of Psychology, University of Jaen, Jaen, Spain

**Keywords:** suicide, resilience, protective factors, prediction, suicide reattempt

## Abstract

The objective of this work was to construct and validate an instrument for assessing resilience to suicide attempts in a Spanish clinical population that has made a previous attempt, and to verify its efficacy for predicting future suicide reattempts at 6 months. For the construction of a Scale of Resilience to Suicide Attempts (SRSA) the theoretical-rational strategy was used. The constructed SRSA-18 consisted of 18 items and 3 subdimensions (internal and external protection and emotional stability), had high internal consistency (α = 0.88; ω = 0.89) and a high positive correlation with the Suicide Resilience Inventory-25, SRI-25 (*r* = 0.91; *p* < 0.01), and to a lesser extent with general resilience scales such as the Connor-Davidson Resilience Scale, CD-RISC (*r* = 0.79; *p* < 0.01) and the Resilience Scale of 14 items, RS-14 (*r* = 0.76; *p* < 0.01). Additionally, a specific SRSA-18 score predicted future suicide reattempts 6 months after the first attempt. This new scale (SRSA-18) assesses in a brief and rapid way, through protective factors rather than risk factors, the level of resilience to the suicide attempt in specific clinical subpopulations in hospital emergency services, being able to prevent suicide reattempts with higher lethality.

## Introduction

The protective factors that promote resilience are diverse and depend on the adverse situation suffered (Johnson et al., 2013). The suicide attempt is a risk behavior, modulated among others by sociocultural aspects, and for which the protective factors that minimize the level of lethality have not been studied in depth. Suicide is a serious global public health problem (World Health Organization (WHO), 2014) and is one of the main causes of death in the world (Nock et al., 2018). One suicide occurs every 40 seconds, and almost 800,000 people die each year from this cause (World Health Organization (WHO), 2016). For every person who dies, 20 attempt it, the suicide attempt being the only behavior that can predict future more lethal reattempts or completed suicide (Posner et al., 2011; World Health Organization (WHO), 2018). The personal and social costs associated with repeated self-harm are very high, but in addition, people who have injured themselves repeatedly are more than twice as likely to die by suicide compared to those who self-injured only once (Zahl and Hawton, 2004; Serafini et al., 2017). Specifically, a suicide attempt can predict future more lethal reattempts or death by suicide, especially between 6 and 12 months after the first attempt (Borges et al., 2010; Chan et al., 2016). However, the epigenetic and multidimensional nature of suicidal behavior makes its prevention difficult (Wasserman et al., 2010), due in part to the fact that repeated behaviors are deeply modulated by risk factors such as sex, age (Sánchez-Teruel et al., 2018), and sociocultural factors (Lopez-Castroman et al., 2015a).

Most studies on suicide attempts or reattempts have focused on risk factors (Arensman et al., 2019; Sher, 2019a) rather than on the protective variables that can minimize suicide reattempt (Larkin et al., 2014; Sánchez-Teruel et al., 2020). Interestingly, there is a significant proportion of people who, after the first attempt, do not make another suicide attempt, not even in adverse situations (Deuter et al., 2020). Some authors have hypothesized that these people can put in place adaptation mechanisms after their first attempt to cope with the risk factors to which they are exposed in the future (Masten, 1999; Sher, 2019b). This process can be determined by the existence of protective factors that interact to minimize the risk factors and can produce different results (Johnson et al., 2017; Masten and Cicchetti, 2016). This line of work is consistent with research on mental health that emphasizes the urgent need to change the focus from psychopathology to resilience (Masten, 2016; Kalisch et al., 2019).

Resilience has important implications for the prevention of psychopathology and the promotion of human development (Collins et al., 2018; Masten, 2019). Low resilience has been shown to be a predictor of risk for suicidal behavior (Kim et al., 2020) and attempts have been made to propose concrete models of resilience-based suicide protection for specific clinical subpopulations (Rutter, 2008). However, a person can be resilient to some specific risk situations, but not to others (Matel-Anderson et al., 2019; Stainton et al., 2019). Hence, it is essential to measure resilience specifically in people who have made suicide attempts, as the factors that protect against suicidal behavior may be different depending on the type of suicidal phase (ideation, attempt, or suicide) and culture of the individual (Siegmann et al., 2018).

Although there are results that support the importance of measuring resilience in the context of suicide attempt (Sher, 2019b), most studies use resilience measurement instruments created from people with symptoms of post-traumatic stress disorder (Connor and Davidson, 2003) and are applied to older people from the general population (Wagnild and Young, 1993) or to people exposed to adverse situations or with a high level of stress (Johnson et al., 2010a; Sánchez-Teruel and Robles-Bello, 2014). Very few studies use specific instruments to measure resilience to suicide in a population with previous suicide attempts, longitudinally assessing this aspect in the months of greatest vulnerability after the first attempt. A frequently used instrument is the Suicide Resilience Inventory-25 (SRI-25) by Osman et al. (2004), designed to measure resilience to suicide in adolescents and young people. However, this instrument assesses suicidal ideation in university students without previous suicide attempts (Rutter et al., 2008), and its items are exclusively based on risk factors. Attempts have been made to verify the factorial structure and psychometric properties of SRI-25 in adolescents admitted to psychiatric hospitals, but the heterogeneity of the sample regarding suicidal behavior has been considered a limitation even by the authors themselves (Gutierrez et al., 2012). Villalobos-Galvis et al. (2012) translated this instrument into Spanish, but they only applied it to young adults in Colombia without previous suicide attempts. Therefore, the suicide attempt would be a culturally modulated behavior (Lopez-Castroman et al., 2015a; Lester et al., 2020) and the protective factors that produce resilience could be different depending on the adverse situation suffered (Masten, 2016, 2019; Sher, 2019a). Considering that the few existing measuring instruments present some structural limitations (Gutierrez et al., 2012), the need to create appropriate instruments to measure resilience, based on protective factors and culturally adapted to the Spanish population with a previous suicide attempt, is evident.

The objective of this work was to construct and validate an instrument for the assessment of resilience to suicide attempts in a Spanish clinical population that has made a previous attempt, and to verify its efficacy for predicting suicide reattempts at 6 months. In addition, the structural validity and convergent and divergent reliability of the instrument were assessed with other resilience measures, as well as its ability to predict suicide reattempts at 6 months and its diagnostic efficacy.

## Materials and Methods

### Participants

The initial sample consisted of 147 participants who had to meet the following inclusion criteria:

(1) Age between 18 and 95 years old
(2) Understand and speak Spanish correctly
(3) Have made a previous suicide attempt
(4) Have been diagnosed by the emergency doctor with “self-harm,” “self-injurious behavior,” or “suicide attempt”
(5) Having been admitted through the emergency services of any of the public or private hospitals in the province of Jaen (Spain)
(6) Have signed the informed consent for their participation in the study. Of the total initial sample, four participants were excluded due to errors or omissions in their responses to the evaluation questionnaires and 12 were excluded due to the impossibility of contacting them by phone during the 6-month follow-up, after discharge from the health care emergency service (second phase described in procedure). The final sample consisted of 131 participants, where 77 (58.7%) were women and 54 (41.3%) were men, with ages between 18 and 73 years (*M* = 39.6; *SD* = 9.7). The characteristics of the sample are summarized in Table 1. A favorable report was obtained from the Research Ethics Committee of the University of the last author and the Health Research Bioethics Committee of the Regional Government of Jaén (Spain).

**TABLE 1:** Summary of sociodemographic data of the sample (*N* = 131).

	N (%)	Contrast statistic	d.f.	Phi
Studies level		1.52^*ns*^	3	0.56
None	15 (11.5)			
Basic	33 (25.1)			
Cycles/bachelor	54 (41.3)			
University	29 (22.1)			
Civil status		8.22*	3	0.62
Single	39 (29.8)			
Married/domestic partner	79 (60.3)			
Separated/divorced	10 (7.6)			
Widow/widower	3 (2.3)			
Who does the participant live with?		9.74**	6	0.77
Alone	12 (9.1)			
Spouse	10 (7.6)			
Children	3 (2.3)			
Spouse/children	67 (51.1)			
Partner	3 (2.3)			
Parents	34 (26.1)			
Other	2 (1.5)			
N° children		3.11^*ns*^	2	0.52
0	54 (41.2)			
1	20 (15.3)			
2 or more	57 (43.5)			
Employment situation		9.65**	2	0.62
Unemployed	98 (74.8)			
Independent	30 (22.9)			
Works for others	3 (2.3)			
Religion		10.83*	3	0.81
Believer	29 (22.1)			
Believer and practitioner	13 (9.9)			
Non-practitioner	56 (42.7)			
Non-believer/atheist/indifferent	33 (25.2)			
Family psychological disorders		2.37^*ns*^	1	0.75
Yes	61 (46.6)			
No	70 (53.4)			
Family suicide attempt		8.37**	1	0.63
Yes	30 (22.9)			
No	101 (77.1)			
Current disorder diagnosis		9.28**	1	0.86
Physical	4 (3.1)			
Psychological	127 (96.9)			
Attempt prior to current		8.91**	2	0.64
0	122 (93.1)			
1	7 (5.3)			
2	2 (1.6)			

Contrast statistic = ξ^2^ = Chi-Square or t student. *p < *0*.05; **p < *0*.*01; d.f., degrees of freedom; Phi, effect size.*

### Instruments

#### Sociodemographic Data Sheet

An ad hoc data sheet was prepared to collect the identification data (name and telephone numbers) and all the data indicated in Table 1.

#### Connor-Davidson Resilience Scale (CD-RISC) (Connor and Davidson, 2003)

This scale was translated and adapted into Spanish by Manzano-García and Ayala (2013). It is made up of 25 items that assess the resilience level on a Likert-type scale (from 0 = not at all agree to 4 = totally agree). The original version of the scale shows adequate internal consistency that coincides with the Spanish version (alpha = 0.89) and has an adequate test-retest reliability (0.79). The internal consistency in the sample of this study through alpha was 0.62 and through omega was 0.63.

#### Wagnild’s Resilience Scale of 14 Elements (RS-14) (Wagnild, 2009)

This scale was translated and adapted into Spanish by Sánchez-Teruel and Robles-Bello (2015). It measures the degree of individual resilience that allows the person to adapt to adverse situations. The cultural adaptation in the Spanish university population shows adequate internal consistency (α = 0.79), but presents a univariate structure (Sánchez-Teruel and Robles-Bello, 2015). The reliability through Cronbach’s alpha in the sample of this study was 0.65 and omega 0.68.

#### Suicide Resilience Inventory-25 (SRI-25) by Osman et al. (2004)

Translated and adapted into Spanish by Villalobos-Galvis et al. (2012). This scale measures resilience to suicidal ideation. The total score ranges from 0 to 75 points, with a cut-off of 57. The Spanish version presents an alpha of 0.92. In this study, a Cronbach’s alpha of 0.77 and omega 0.79 was obtained.

#### Monthly Telephone Interview for 6 Months (Second Phase)

From the date of discharge in the Emergency Department, monthly interviews were conducted with each participant. They were asked how they were doing, if there had been any adverse situations (none, related to the environment of a partner or ex-partner, related to the family environment, related to the economy, work or studies, death of a close person, others) and how these situations had affected them. Finally, if there had been real suicide attempts since the last occasion (in those cases where a high level of vulnerability was detected, this last question was asked to the family member).

#### Scale of Resilience to Suicide Attempts (SRSA-18) (Supplementary 1)

This scale was made in this study and it is described in the following sections.

### Procedure

For the construction of the SRSA, the theoretical-rational strategy was used, which states that the items are selected according to the most abundant literature on the subject (Bermúdez, 2003; Muñiz et al., 2005). Throughout the entire process, the regulations for the creation of new psychological assessment instruments in clinical population were followed (Wilson, 2005; Hernández et al., 2016; Muñiz and Fonseca, 2017). The start of the SRSA emerged from a review of the literature on resilience to suicide attempts. A review of Pubmed, Psycinfo, Medline, Psicodoc, and Psyke databases was performed with the following inclusion criteria:

(1) articles published by a peer-reviewed journal in Spanish or English
(2) articles published between the years 1980 and 2012, as it was precisely in the eighties when empirical studies related to resilience began to appear (Rutter, 1987)
(3) that the articles used the terms “Resilience-Resiliencia” or “protective factors-factores de protección” as keywords, always combined with “suicide attempt-tentativa suicida”
(4) that the methodology used in the articles was empirical and based on the interaction between two or more variables to result in the suicide attempt (using ANOVA or regression analysis)
(5) that any of the variables included in the study was a psychological construct, understood as any cognitive, emotional or behavioral concept, excluding socio-demographic variables. This process allowed identifying the variables on which the SRSA was based, resulting in an inventory of 70 items with five response options (0 = never; 1 = sometimes; 2 = half of the time; 3 = almost always, 4 = always) (Muñiz et al., 2005).

Subsequently, re-evaluation processes were carried out together with four judges who were experts in resilience and suicide (psychologists) and nine people who had made a suicide attempt (Wilson, 2005). Psychologists tried to analyses whether the item corresponded to the psychological construct or not, and people who had made previous suicide attempts based on their own experience assessed whether this psychosocial aspect was significant or not. This allowed the elimination of some items that had a consensus of less than 85%, and led to a new version of the SRSA in which the number of items was reduced to 34. Subsequently, a comprehension analysis of these 34 items was carried out with a subsample of 18 people who had made suicide attempts, eliminating after this process all those items that were difficult to understand (less than 0.40 for item difficulty index) and whose corrected item-total correlation index did not exceed 0.30 (Haladyna and Rodríguez, 2013). As a result of this process, 16 items were discarded. Next, other assessment instruments were selected to test the validity of the SRSA (Muñiz and Fonseca, 2017). The final version of the instrument consisted of 18 items, and was therefore called SRSA-18 (Muñiz and Fonseca-Pedrero, 2019).

Finally, all the questionnaires were applied to people who had made a suicide attempt during their stay in the emergency services or during hospital admission. In the second phase, telephone interviews were conducted with each participant for 6 months (one interview every 30 days approximately).

### Data Analysis

Incomplete data represented less than 2% of responses, and for these a multiple imputation method (SPSS) was used for missing values (Graham, 2012). Internal consistency tests and item analysis were performed. Next, a confirmatory factor analysis (CFA) was performed with SPSS 23 AMOS (IBM Corporation, 2013) to confirm the structure of SRSA-18. The method used in the confirmatory analysis was the generalized least squares (GLS). The fit indices used were χ^2^/df, the root mean square error of approximation (RMSEA), the comparative fit index (CFI), and the Tucker-Lewis index (TLI). The goodness of the fit model was considered satisfactory when the TLI and the CFI were ≥ 0.95, and the RMSEA approached 0.06 (Kline, 2015). Reliability was also assessed using the internal consistency procedure (Cronbach’s alpha and McDonald’s omega coefficients). Subsequently, external evidence of the validity of the scale (criterion validity) was obtained through the correlation with measures of general resilience (CD-RISC and RS-14) and with measures of resilience to suicidal ideation (SRI-25). Finally, the predictive validity for suicide reattempts in the clinical population was calculated and it was verified whether the prediction made by this scale better predicted suicide reattempts than other scales adapted to the general population. The level of statistical significance required in all tests was a minimum of *p* < 0.05.

## Results

### Descriptive and Item Analysis

The results showed an important variability in the asymmetry and kurtosis of the sample (Table 2), which is indicative of a lack of univariate normality. The item-total correlations were adequate (r item-total > 0.50) and the total Cronbach’s alpha did not improve if any of the items were removed.

**TABLE 2:** Descriptive statistics, asymmetry, kurtosis, and item analysis.

SRSA-18	M (SD)	*K-S*	*A*	*K*	r item-total	α item removed
	
			*TE* (0.21)	*TE* (0.42)		
Item 1	1.02 (1.13)	0.90**	0.17	–0.72	0.67	0.45
Item 2	1.06 (1.28)	0.81**	–0.14	–1.11	0.51	0.47
Item 3	1.07 (1.28)	0.86**	–0.05	1.01	0.35	0.42
Item 4	1.05 (1.15)	0.86*	0.20	–1.02	0.55	0.53
Item 5	1.89 (1.10)	0.85*	0.20	–0.87	0.72	0.52
Item 6	1.05 (1.99)	0.97**	0.06	–0.78	0.61	0.50
Item 7	1.24 (1.94)	0.83**	–0.06	–0.89	0.57	0.64
Item 8	1.04 (1.78)	0.86**	0.07	–0.78	0.53	0.48
Item 9	1.07 (1.45)	0.80**	0.03	–0.77	0.73	0.59
Item 10	1.08 (1.20)	0.87**	–0.01	0.18	0.62	0.51
Item 11	1.04 (1.19)	0.90**	0.07	–0.83	0.79	0.53
Item 12	1.22 (1.95)	0.87**	0.09	–0.81	0.71	0.36
Item 13	1.30 (1.12)	0.91*	–0.03	–0.92	0.51	0.63
Item 14	1.08 (1.11)	0.80**	0.05	–0.86	0.76	0.35
Item 15	1.24 (1.29)	0.55*	–0.10	–1.07	0.58	0.58
Item 16	1.12 (1.96)	0.87**	0.10	–1.12	0.71	0.42
Item 17	1.05 (1.24)	0.89**	–0.09	–0.92	0.62	0.61
Item 18	1.14 (1.86)	0.90**	–0.08	–0.97	0.57	0.52
Total	28.15 (16.21)	0.11**	–0.12	0.19	1	0.82

M, Mean; SD, Standard Deviation; A, Asymmetry; C, Kurtosis; TE, Typical error of asymmetry and kurtosis; K-S, Kolmogorov-Smirnov; *significant correlation at the 0.05 level (bilateral); **significant correlation at the 0.01 level (bilateral).

### Confirmatory Factor Analysis of the SRSA-18

The results of the normality analysis showed that there was no multivariate normality (Mardia = 437.51). The ratio χ^2^/df obtained significant values lower than 3, showing a good fit. The Residual Mean Square-RMR obtained an acceptable fit with values equal to or less than 0.08. The mean square error of approximation (RMSEA-95% CI) was 0.03, which indicates an excellent fit. The CFI, TLI, and GFI values were greater than 0.95, which also indicates a good fit of the data (Table 3).

**TABLE 3:** Goodness-of-fit indices of the confirmatory factor analysis (CFA).

	χ^2^	df	χ^2^/gl	p	RMSEA (IC 95%)	RMR	CFI	TLI	GFI
SRSA-18	129.35	32	2.51	0.00	0.03 [0.01; 0.04]	0.08	0.98	0.97	0.95

**χ^2^, Chi square; df, degrees of freedom, χ^2^/gl, Chi square goodness of fit index; p, significance level; RMSEA, Root mean square error of approximation; CFI, Comparative fit index; TLI, Tucker-Lewis index; RMR, Root mean residue; GFI, Gamma index.**

**FIGURE 1:**
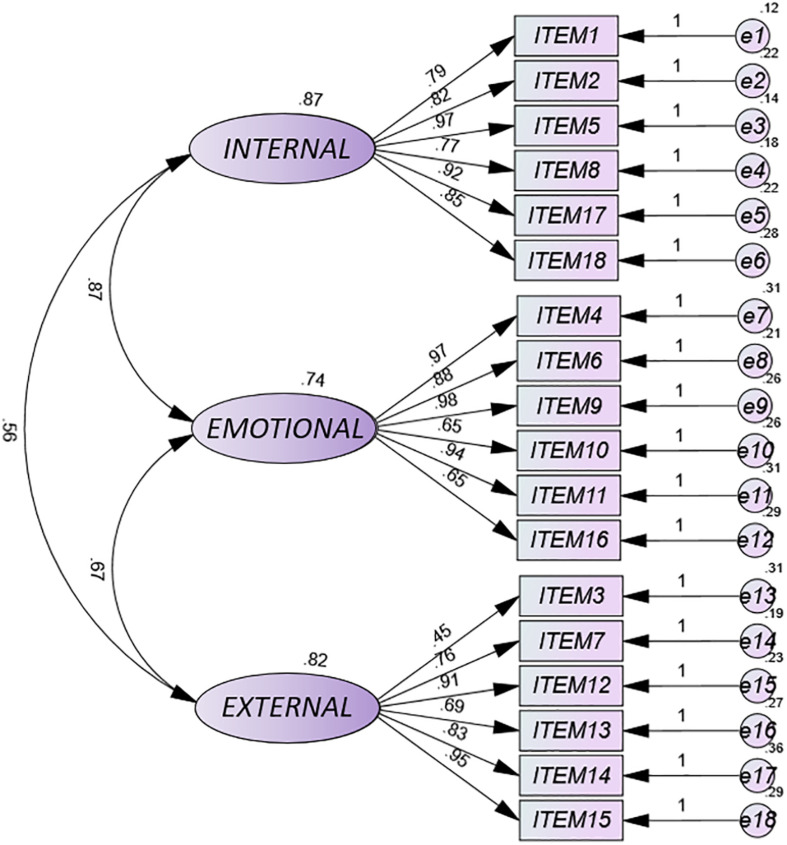
Path diagram of SRSA-18.

Figure 1 shows the path diagram of the SRSA-18 in the sample of people who made a suicide attempt, with all the values of the standardized weights (beta coefficients, β) greater than 0.30. The factor loadings for each item in its respective dimension (internal protection, external protection and emotional stability) were found between high and very high. Specifically and globally, the results showed that the item with the lowest weight was item 3 (0.45) (external protection) and the item with the greatest weight was item 9 (0.98) (emotional stability). On the other hand, the lowest covariances were found between the internal and external dimensions (0.56), and the highest between the internal dimension and emotional stability (0.87). The analysis of results by dimensions showed that the item with the lowest factorial load in the internal protection dimension was item 8 (0.77) “I am as good at what I do as my colleagues or friends,” and the item with the highest factorial load in the internal dimension was item 5 (0.97) “I take problems with humor.” In the emotional stability dimension, the items with the lowest factor loadings were items 10 (0.65) “I am able to control my anger” and 16 (0.65) “I control my impulses, even if I am pressured”; while the item with the greatest weight was item 9 “I hope to have a happy life” (0.98). Finally, in the external dimension, the item with the lowest factorial load was item 3 (0.45) “If I have a problem, I ask my family or friends for help” and the item with the highest factorial load was item 15 (0.95) “When something worries me I have people who comfort me, listen and encourage me.”

### Reliability Measured Through Internal Consistency and Two Halves for SRSA-18

The internal consistency of the three dimensions and the total score of the SRSA-18 were calculated using the alpha and omega coefficients. On the other hand, two-halves reliability was calculated, both for each dimension and for the full scale (18 items), also obtaining acceptable coefficients (Table 4).

**TABLE 4:** Internal consistency (alpha, omega, and two-halves) for SRSA-18 and by dimensions.

Dimensions	M	SD	*A*	*K*	*K-S*	r_*xx*_	α	ω
	
			*TE* (0.13)	*TE* (0.22)				
Internal protection	12.29	4.41	–0.20	–1.14	0.71*	0.73	0.81	0.87
Emotional stability	12.61	2.52	–0.13	–1.06	0.99**	0.59	0.79	0.82
External protection	10.21	5.57	–0.16	–1.09	0.23*	0.69	0.71	0.78
Total	21.02	11.98	0.92	–1.12	0.98**	0.76	0.88	0.89

M, Mean; SD, Standard Deviation; A, Asymmetry; K, Kurtosis; TE, Typical error of asymmetry and kurtosis; K-S, Kolmogorov-Smirnov test; *significant < 0.05; **significant < 0.01; ns, not significant; rxx, Two halves reliability; α, alpha; ω, Omega coefficient.

### Convergent and Divergent Validity of the SRSA-18

To determine if the SRSA-18 assesses resilience to suicidal attempts, the relationship between the SRSA-18 and other instruments used to assess general resilience (CD-RISC and RS-14) and resilience to suicidal ideation (SRI-25) was verified. Regarding convergent validity, Table 5 shows that the total score of the SRSA-18 presented a high positive correlation with all the instruments used, being the highest correlation with the total score of the SRI-25. Regarding SRSA-18 dimensions, the internal protection dimension showed high positive correlations with the SRI-25 scale, as did the emotional stability and external protection dimension. The correlation levels of the three dimensions of the SRSA-18 with the general resilience scales (CD-RISC and RS-14) were lower.

**TABLE 5:** Correlations between SRSA-18 and subdimensions with CD-RISC, RS-14, SRI-25.

	*IP*	*ES*	*EP*	SRSA-18	CD-RISC	RS-14	SRI-25
*IP*	1	0.90**	0.79**	0.95**	0.61*	0.67**	0.84**
*ES*	0.90**	1	0.83**	0.96**	0.53*	0.59**	0.83**
*EP*	0.79**	0.83**	1	0.92**	0.58*	0.63**	0.77**
ERATS-18	0.95**	0.96**	0.92**	1	0.79*	0.76**	0.91**
CD-RISC	0.61*	0.53*	0.58*	0.79*	1	0.93**	0.95**
RS-14	0.67**	0.59**	0.63**	0.76**	0.93**	1	0.93**
SRI-25	0.84**	0.83**	0.77**	0.91**	0.95**	0.93**	1

IP, Internal protection, ES, Emotional stability; EP, External protection; *significant correlation at level 0.05; **significant correlation at level 0.01.

### Prediction of Suicide Reattempts and Analysis of Diagnostic Efficacy

Initial results showed a significant difference in the mean and standard deviation of the total SRSA-18 scores between people who have made a suicide reattempt and people who have not made a suicide reattempt during the 6-month period of follow-up, the former having a lower score than the latter. Initially, the appropriateness assumptions of binary logistic regression analysis were calculated to quantify the predictive power of SRSA-18 as an independent variable (IV) on monthly suicide reattempt (Yes/No) (dependent variable, DV) over 6 months. Preliminary analyzes for the evaluation of goodness of fit confirmed that the non-multicollinearity assumptions were fulfilled (<5, PIV = 1.00 and 1.77; Kleinbaum et al., 1988) and that the tolerance values (1–0.1) were between 1 and 0.98 (Lomax and Hahs-Vaughn, 2012). Furthermore, there was no autocorrelation in any of the variables, so the assumption of independence of the error was fulfilled (Durbin-Watson = 1–3) and the results can be generalized to the general population, with a maximum coefficient of 2.11 (D-W = 1.86–2.11) (Yoo et al., 2014; Table 6).

**TABLE 6:** Predictive model of the SRSA-18 for 6-month follow-up period.

Month	D-W	χ^2^	% I/P	R^2^N	B	SE	Wald	Exp(β)	I.C. (95%) para Exp(β)	
	
									LL	UL
1°	2.03	5.95*	87.21/98.51	0.175	–0.011	0.09	1.40*	0.90	0.75	1.08
2^a^	1.86	9.78**	72.90/95.42	0.209	–0.20	0.08	5.73*	0.82	0.70	0.97
3°	2.11	3.71*	93.27/97.13	0.316	–0.08	0.05	3.32*	0.92	0.84	1.01
4°	2.09	13.51**	67.20/72.43	0.316	–0.27	0.14	3.59**	0.76	0.57	1.01
5^a^	1.99	11.96**	62.41/85.41	0.701	–0.15	0.05	9.46**	0.86	0.78	0.95
6°	1.92	11.83**	63.12/89.39	0.782	–0.11	0.03	11.41**	0.90	0.84	0.96

D-W, Durwin-Watson test; χ^2^, ROA statistical efficiency test with 1 degree of freedom; % I/P, global percentage observed and global percentage; *significant correlation at the 0.05 level; **significant correlation at the 0.01 level; ns, not significant; R2 Nagelkerke, Variance explained by IV; β, Beta coefficient; SE, Standard error; Wald, Contrast power statistic; Exp(β), Result of the regression equation: LL, Lower limit; UP, Upper limit.

On the other hand, the significant result given by the ROA statistical efficiency score (χ^2^; *p* < 0.05) indicated that there is an improvement in the prediction of the probability of occurrence of the DV categories (suicide reattempt or no suicide reattempt) during the follow-up months, with an increase in the probability of success in the DV result when the IV has a low score (SRSA-18). The value of R2 Nagelkerke (part of the variance explained by the DV) indicated that the model proposed for the SRSA-18 explains between 17.5 and 78.2% of the variance of the DV according to each month (Table 6). The results indicated that the predictive power of this model could be tested, as the IV that has been introduced in the model significantly improved the prediction (initial percentage and predicted percentage); and the models explained 78.2% of the variance of the DV in the sixth month. The Wald score indicated that the SRSA-18 contributes a significant value to the prediction of a suicide reattempt (*p* < 0.05). Furthermore, the Wald index showed that the results can be generalized to samples of people who have made a suicide attempt using the SRSA-18 score (1.40–11.41). Likewise, the results of the EXP(β) regression equation indicated that for people who have made a suicide attempt [<1 of the EXP(β); Kleinbaum et al., 1988; De Maris, 2002], the probability of a suicide reattempt would increase, at least during the first 6 months after the first attempt.

**FIGURE 2:**
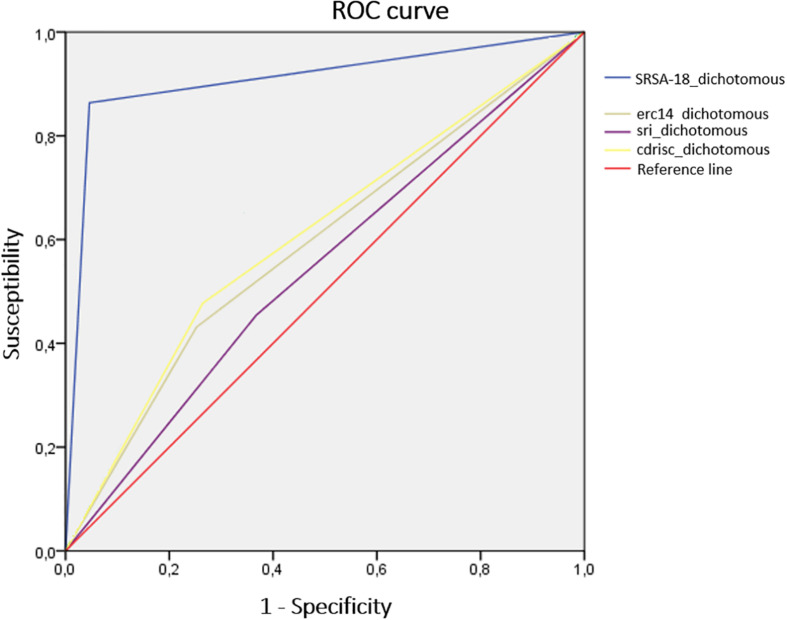
Area under the ROC curve of dichotomized resilience.

Regarding the diagnostic efficacy of the SRSA-18 and the other instruments used, given that a third of the subjects in the clinical sample attempted suicide during the 6-month follow-up period (44 of the 131), it was decided to use this cut-off point for the SRSA-18 and for the rest of the instruments. Thus, the 33rd centile coincided with the 18 score of the SRSA-18. For the CD-RISC scale, the score corresponding to the 33rd centile was 42 points; 49 for the SRI-25 and 45 for the RS-14. In this way, any score above that cut-off point would be considered high resilience and, conversely, a score below that cut-off point would be considered low resilience. If these scales are dichotomized, they can be used as predictors of suicide reattempts during follow-up. In order to analyze the predictive efficacy of each of these dichotomized instruments in the classification of subjects who would or would not attempt suicide during the 6-month follow-up, the areas under the ROC (Receiver Operating Characteristic) curves of each instrument were calculated, as well as other indices of diagnostic efficacy such as sensitivity and specificity (Carvajal et al., 2011). Other cut-off points in addition to the one that matched the 33rd centile were tested to see if results could be improved with other thresholds. Only on the SRSA-18, the best indices coincided with the 33rd centile (direct score: 18). In the other instruments, the cut-off point had to be changed to obtain an improvement in the indices that assess the quality of the instruments’ prediction. Thus, the cut-off points for the other instruments (and their centile) were: CD-RISC = 43 (34th centile), SRI-25 = 52 (40th centile); ER-14 = 43 (29th centile). The areas under the ROC curve of each instrument (Figure 2), as well as the Odds Ratio (OR), the sensitivity and specificity of all these measures are shown in Table 7.

**TABLE 7:** Efficiency in the prediction of each instrument.

Index	SRSA-18	CD-RISC	SRI-25	RS-14
Sensitivity	0.86	0.48	0.46	0.43
Specificity	0.95	0.74	0.63	0.75
Odds ratio	131.42	2.5	1.4	2.2
Area under the ROC curve	0.91	0.61	0.54	0.59
Significance	<0.001	<0.05	ns	ns
Confidence interval (95%)	0.84–0.97	0.51–0.71	0.44–0.65	0.49–0.7

## Discussion

The factor analysis confirmed the three-dimensional structure of the scale (internal and external protection and emotional stability). The internal consistency of the total SRSA-18 in this sample was high both in the total score and in each of the subdimensions, these results agreeing with other studies on this construct (Gutierrez et al., 2012). This result was expected, as the scale was based on the most relevant literature results on protective factors that modulate resilience to a specific risk behavior (suicide attempt). Other studies have obtained low levels for alpha and omega in other general resilience scales such as the CD-RISC (Connor and Davidson, 2003) or the RS-14 (Wagnild, 2009), and a moderate level of alpha when related to the SRI-25 scale (Osman et al., 2004). This fact can be explained because in this study we used a population sample with previous suicide attempts, whereas Osman et al. (2004) used a sample of young people and university students in which suicidal ideation was used as the outcome measure. This shows that different assessments of resilience are obtained according to the different severity of the result (ideation vs. suicide attempt). In addition, the correlations of the total SRSA-18 and its dimensions with instruments of general resilience (CD-RISC, ER-14) and of resilience to suicidal ideation (SRI-25), presented a positive and significant correlation (*p* < 0.05), being the highest correlation with the total score of the SRI-25 (*r* = 0.91; *p* < 0.01). Both scales (CD-RISC and RS-14) measure general resilience while the SRSA-18 assesses specific resilience to a specific risk (suicide attempt). This same explanation could respond to the high correlations that the SRSA-18 presented with the SRI-25, as within the continuum of suicidal behavior, ideation (SRI-25) and suicide attempt (SRSA-18) are related. Additionally, in comparison with the SRI-25, the SRSA-18 does not indicate words or phrases related to suicide in any of its items, while the SRI-25 does. This would indicate that the SRSA-18 measures suicide attempts in an indirect way, focusing more on protective factors than risk factors, which really defines resilience as a result, as other recent studies on resilience have proposed (Masten, 2019; Sher, 2019a).

A score of less than 18 points on the SRSA-18 predicts a suicide reattempt within 6 months of a first attempt. That is, if there is a decrease in the score obtained on the SRSA-18 in people who have already made a suicide attempt, the probability of a suicide reattempt increases during at least the first 6 months after discharge from the emergency service. The SRSA-18 showed high specificity and sensitivity, obtaining an 86% probability that a person will make a suicide reattempt and a 95% probability of detecting people who will not make a reattempt in the 6 months after the first suicide attempt if their direct score is 18 or less.

A surprising fact is that the only instrument that assesses resilience to suicidal ideas (SRI-25) is the one that presented the worst indexes of specificity and sensitivity and area under the curve compared to the SRSA-18. The measurement of resilience to different outcomes (ideation in SRI-25 vs. attempt in SRSA-18) in suicide could be the basis of the predictive discrepancies between both scales. This hypothesis is confirmed by previous studies on the specific clinical typology of the attempt compared to other phases of suicide such as ideation (Johnson et al., 2010b; Mustanski and Liu, 2013), self-inflicted injuries (Christiansen and Larsen, 2012), or planning (McLean et al., 2008; Nock et al., 2018).

The SRSA-18 focuses on assessing protective factors rather than risk factors. This characteristic is extremely important because it gives it a new aspect, as it is the only existing scale that assesses resilience to suicide attempt based on protective factors. It also shows that below 18 points there may be a high risk of suicide reattempt, this second time more lethal. Additionally, the SRSA-18 has a small number of items (18) and has been adapted and validated in a Spanish population with previous suicide attempts, which could facilitate its application in various contexts such as research or mental health on suicide attempts in Spain. The results of this work are in line with the results of other studies (Johnson et al., 2010a; Lopez-Castroman et al., 2015b), and confirm the need to validate suicide assessment instruments adapted to the clinical population so that their risk assessment levels are adequate (Ayuso-Mateos et al., 2012; World Health Organization (WHO), 2016), as well as to carry out validations and adaptations of the instruments to the target population of the country of origin, due to the tremendous cultural differences that may exist, even in areas with a similar language (Alcorta-Garza et al., 2016). Also, this study opens up new lines of work to promote specific treatments to increase protective factors in people who have already made a suicide attempt.

The main contribution of this research work is that it has been found that people who have attempted suicide who have a direct score of less than 18 points are likely to do it again, while those who scored more than 18 points were not likely to attempt it again. Therefore, the implications at a predictive level are very interesting for preventive work.

However, some limitations must be mentioned. In the first place, the sample consisted mostly of women, so it would be appropriate to homogenize this variable, this makes sense because the scientific literature recalls that suicide attempts are more frequent in men than in women, however, deaths are more frequent in the case of men (World Health Organization (WHO), 2018). A second important limitation was the small number of people who had made a suicide attempt (*N* = 131); it would be advisable in subsequent studies to validate the SRSA-18 in larger samples, with respect to the small sample size, it must be considered that this research was conducted with a clinical population in a situation of attempted suicide in a hospital emergency room, which makes the situation very complex and prioritizes the physical recovery of the participants, with the psychosocial tests being secondary. Third, the territorial contextualization of the data obtained also made it difficult to generalize the results, in any case, the data would only be generalizable in a similar cultural context of medium and high per capita income. Lastly, the 6-month follow-up period was short, so it would be advisable to propose broader follow-up processes to check if the prediction of suicide attempts is really fulfilled through the instrument created, beyond the 6 months valued in this study.

## Data Availability Statement

The raw data supporting the conclusions of this article will be made available by the authors, without undue reservation.

## Ethics Statement

All procedures performed involving human participants were in accordance with the ethical standards of the institutional research committee and with the 1964 Helsinki declaration and its later amendments or comparable ethical standards. This manuscript has a favorable report from Bioethics Commission of the University of Jaen, Spain with CEIH Number 011113-3b. The patients/participants provided their written informed consent to participate in this study.

## Author Contributions

DS-T: conceptualization, writing—original draft preparation, supervision, experimentation, modeling validation, investigation, modeling reviewing, methodology, reviewing, and editing. MR-B: writing—original draft preparation and supervision. JM-M: supervision, experimentation, investigation, and modeling reviewing. AG-L: methodology, writing—reviewing, and editing. All authors contributed to the article and approved the submitted version.

## Conflict of Interest

The authors declare that the research was conducted in the absence of any commercial or financial relationships that could be construed as a potential conflict of interest.
