# Supplementary material for: Resilience Assessment Scale for the Prediction of Suicide Reattempt in Clinical Population
Source: Front Psychol. 2021 May 13;12:673088. doi: 10.3389/fpsyg.2021.673088 (PMC8155352; doi:10.3389/fpsyg.2021.673088)
Supplement: Supplementary file 1 [file Table_1.DOCX]

Supplementary Material

## Scale of Resilience to Suicide Attempts, SRSA-18

This scale pretends to know some aspects of your life. Answer all questions by marking an (x) in the number, whichever you think best applies to your situation.

|  | **Never** | **Sometimes** | **Half of the time** | **Almost always** | **Always** |
| --- | --- | --- | --- | --- | --- |
| 1. I always see the glass half full, instead of half empty | 0 | 1 | 2 | 3 | 4 |
| 2. I am a valuable person | 0 | 1 | 2 | 3 | 4 |
| 3. If I have a problem, I ask my family or friends for help | 0 | 1 | 2 | 3 | 4 |
| 4. I have plans for the future | 0 | 1 | 2 | 3 | 4 |
| 5. I take problems with humor | 0 | 1 | 2 | 3 | 4 |
| 6. Emotions don't get over me | 0 | 1 | 2 | 3 | 4 |
| 7. I make friends easily | 0 | 1 | 2 | 3 | 4 |
| 8. I am as good at what I do as my colleagues or friends | 0 | 1 | 2 | 3 | 4 |
| 9. I hope to have a happy life | 0 | 1 | 2 | 3 | 4 |
| 10. I am able to control my anger | 0 | 1 | 2 | 3 | 4 |
| 11. I often think before I act | 0 | 1 | 2 | 3 | 4 |
| 12. There are people who are interested in me and what happens to me | 0 | 1 | 2 | 3 | 4 |
| 13. I am able to share my problems with family or friends | 0 | 1 | 2 | 3 | 4 |
| 14. I have a group of friends to have fun | 0 | 1 | 2 | 3 | 4 |
| 15. When something worries me I have people who comfort me, listen and encourage me | 0 | 1 | 2 | 3 | 4 |
| 16. I control my impulses, even if I am pressured | 0 | 1 | 2 | 3 | 4 |
| 17. I know how to get the funny part out of problems | 0 | 1 | 2 | 3 | 4 |
| 18. In tough times I usually hope for the best | 0 | 1 | 2 | 3 | 4 |
